# Transfer of statistical learning from speech perception to production generalizes to reading

**DOI:** 10.3758/s13423-026-02874-y

**Published:** 2026-03-04

**Authors:** Kyle D. Huffaker, Lori L. Holt, Nazbanou Nozari

**Affiliations:** 1 Department of Psychological and Brain Sciences, Indiana University, 1101 E 10th St., Bloomington, IN 47405 USA; 2 Department of Psychology, University of Texas at Austin, Austin, TX USA; 3 Cognitive Science Program, Indiana University, Bloomington, IN USA

**Keywords:** Speech perception, Speech production, Statistical learning, Phonetic convergence, Reading

## Abstract

Past research has shown that short-term exposure to speech carrying certain acoustic statistics transfers robustly to speech production. However, all studies reporting such transfer have used auditory repetition tasks. Therefore, it is unclear whether perception-production transfer in the acoustic-phonetic domain extends to tasks without an auditory model to probe production. We answer this question in two experiments. Experiment 1 shows that people read aloud the words BEER and PEER differently after exposure to auditory samples of “beer” and “peer” drawn from a distribution of standard American English versus a distribution of slightly accented speech. Experiments 2A and 2B replicate this finding and show generalization to reading a new word pair (BEACH/PEACH) and a new nonword pair (BEETH/PEETH). Collectively, these results demonstrate that the perception-production transfer in the acoustic-phonetic domain extends beyond auditory repetition tasks to production tasks without an explicit auditory model, and that this transfer generalizes to new syllables.

**Supplementary Information:**

The online version contains supplementary material available at 10.3758/s13423-026-02874-y.

## Introduction

When interacting with speakers with an accent, we sometimes find ourselves speaking like them (Pardo et al., 2017). Despite a lifetime’s experience speaking in our native accent, the speech patterns of an interlocutor can rapidly reshape our own (Murphy et al., 2024, 2025a, 2025b). For example, Murphy et al. (2024) showed that exposure to slightly accented “beer”/“pier” utterances swiftly changed how listeners produced those words. In American English, /b/ is typically produced with a short voice onset time (VOT) and low fundamental frequency (F0), while /p/ is produced with a long VOT and high F0. A slight accent can be created by reversing these correlations, i.e., shifting /b/ signaled by short VOT to possess a high F0, and a /p/ with long VOT and a low F0 (Idemaru & Holt, 2011). Because VOT is a stronger acoustic cue than F0, listeners exposed to such reverse statistics still hear a /b/ as a /b/ and a /p/ as a /p/ but implicitly *downweight* F0 as an informative cue for the phoneme category. This is evident in the change to categorizing auditory stimuli when VOT is no longer an informative cue: When tested with VOT-ambiguous “beer”/“pier” syllables after listening to a typical American English accent, listeners to tend to report the stimuli with a low F0 as “beer” and those with a high F0 as “pier.” However, after exposure to the accented speech, F0 no longer distinguishes between these two syllables, leading listeners to categorize F0-differentiated stimuli equally as /b/ and /p/ (Idemaru & Holt, 2011; Hodson et al., 2023). Murphy et al. (2024) extended this finding to production by asking listeners to repeat the test syllable. They found that, in line with the downweighting of F0 in perception, the difference in production F0s for “pier” and “beer” was reduced after exposure to accented speech. This finding was replicated in Murphy et al. (2025a, 2025b).

While the above studies show a robust transfer of changes from perception to production, all of them have used auditory repetition tasks that require the speaker to model an auditory probe. A reasonable criticism of such studies is that the claim of transfer to “production” is overstated, as auditory repetition is not a pure production task. Importantly, it could be carried out by direct mapping of input to output phonology, bypassing lexical representations and the process of mapping those representations to their phonemes (Nozari et al., 2010; Nozari & Dell, 2013). It thus remains an open question: Is perception-production transfer also observed in production tasks that do not involve the auditory perception of a test stimulus? This paper answers this question by eliciting production through reading.

Examining transfer in the context of reading helps further isolate the cognitive component of perception-production transfer. In prior studies using the auditory repetition paradigm, both the exposure and the test stimuli carried some social information, such as the speaker’s sex. A reading paradigm removes all social cues from the test stimulus, as it appears in text format. Such cues are known to modulate the convergence of one’s speech to the interlocutor’s speech. For example, convergence can vary as a function of the match or mismatch between the listener’s and the interlocutor’s gender (Miller et al., 2010; Pardo et al., 2017). Similarly, a host of social factors, such as the speaker’s perceived ethnicity, region, and social status can alter convergence (e.g., Bourhis & Giles, 1977; Giles et al., 1991; Pardo, 2006).

An additional advantage of reading over auditory repetition is the examination of generalization to syllables that were never heard in the study. Generalization is often assessed by exposing listeners to one pair (e.g., “bear”, “pear”) and testing them on a different pair (e.g., “beer”, “pier”). By manipulating the overlap between the exposure and test pair, we can determine the representations involved in downweighting in perception and their transfer to production. Murphy et al. (2025b) tested two levels of generalization: phonemic and sub-phonemic. In the phonemic generalization task, the critical phonemes (i.e., /b/ and /p/) were shared between the exposure and test pairs, but the vowel was different (e.g., bear/pear → beer/pier). Uncovering generalization at this level implies that the effects do not require overlap in full words, syllables, or even a CV; rather, having the same phoneme is sufficient to reproduce the effect. In subphonemic generalization, the exposure and test pairs did not share the critical phonemes but shared the same critical dimensions (VOT and F0) and their correlations. For example, if the exposure pair contained /b/ and /p/ (e.g., “bear/pear”), the test pair contained /d/ and /t/ (e.g., “dear/tear”), where /d/ and /t/ have the same relationship to one another regarding VOT and F0 as /b/ and /p/. Uncovering generalization at this level implies that the effects do not depend on phonemic categories, but rather on the change to the representation of acoustic dimensions themselves.

In line with past studies (Idemaru & Holt, 2014, 2020), Murphy et al. (2025b) found generalization at the phonemic, but not subphonemic, level in perception. However, they did not find generalization to production at either level. One interpretation of this finding is that critical units of processing may be different in perception and production (Samuel, 2020). However, Murphy et al. (2025b) did not test CV-level generalization, where the exposure and test syllables share the CV- but not the coda C. Given coarticulation, production may show generalization across syllables, as long as the CV is shared between exposure and test pairs. Therefore, the current study targeted CV-level generalization in reading both words and nonwords.

The rationale for including both words and nonwords as test materials was twofold. First, nonwords provide a robust test of CV-level generalization from learning across word stimuli in perception. For example, when the sort of perceptual downweighting studied by Murphy et al. (2025b) is elicited by word stimuli, it readily generalizes to nonsense syllables with the same phoneme onsets (Lehet & Holt, 2020; Liu & Holt, 2015; see also Kraljic & Samuel, 2005, for an example in another paradigm). Second, and more importantly, words and nonwords could tap into different cognitive systems during reading. According to the dual-route cascaded (DRC) model of reading (Coltheart et al., 2001; Coltheart, 2006), words could be read using a lexical route (which may or may not reach semantic knowledge). In this route, visual features of letters activate letter representations, and those, in turn, activate the word’s orthographic representation in the lexicon. The orthographic representation then activates its corresponding phonological representation in the phonological lexicon, which then activates its corresponding phonemes. Nonwords, on the other hand, cannot be read this way because they do not have any stored representations in the orthographic or the phonological lexicon. DRC, thus, proposes a graphic-phoneme correspondence (GPC) route for reading nonwords. Rather than relying on stored representations in the lexicon, this route uses rules to convert letter strings directly into phoneme strings. Note that words could also be read through the GPC as long as they have regular spelling; however, reading through the lexical route is faster and more efficient.

The GPC route is close to the nonlexical route of auditory word repetition (Nozari et al., 2010), which directly maps input to output phonology, bypassing lexical representations. Here, too, lexical items can be produced using either route, although using the nonlexical route alone for repeating lexical items is uncommon (Nozari & Dell, 2013). Prior studies of perception-production transfer cannot disentangle the involvement of lexical versus nonlexical route in transfer, because nonwords were not used in those studies. But understanding this issue is important in predicting the scope of the effect. It is possible that the perception-production effect depends on direct mapping of input to output phonology. Prior findings on word pairs should then be interpreted as them having been repeated primarily (although most likely not exclusively) through the nonlexical route, thus excluding the involvement of their stored lexical representation in production. If true, then any generalization effects are expected to be stronger for nonwords, which exclusively use this route, compared to words. This possibility is theoretically interesting, but it decidedly limits the scope of transfer, as everyday speaking does not generally involve bypassing stored lexical representations. On the other hand, if transfer is present even when lexical representations are engaged, transfer should not show a large advantage for nonwords.

In summary, the current study had two goals: (a) to examine the basic perception-production transfer in reading, which does not have an auditory model (Experiment 1), and if uncovered, (b) to test generalization of such transfer to words and nonwords without participants having heard an auditory model for either (Experiments 2A, B).

## Experiment 1

### Method

#### Participants

A power analysis (PANGEA; Westfall, 2015) showed that, to detect a Condition × First Letter interaction with an effect size of 0.3 as the smallest effect size of interest, with a power of 0.8 and alpha of 0.05, a sample size of 33 was required. Accordingly, we recruited 44 participants using Prolific (www.prolific.com) to allow for attrition. Participants were adult English speakers in the USA, aged 18–35 years with initial English exposure before age 2 years. All reported normal hearing. They were paid $10/h for their time. Eleven participants were rejected due to background noise in recordings preventing F0 extraction or failure to comply with experimental instructions. Data from 33 participants (*M*_Age_ = 28.6, *SD* = 5 years; *N*_Female_ = 16) entered the analysis.

#### Stimuli

**Fig. 1:**
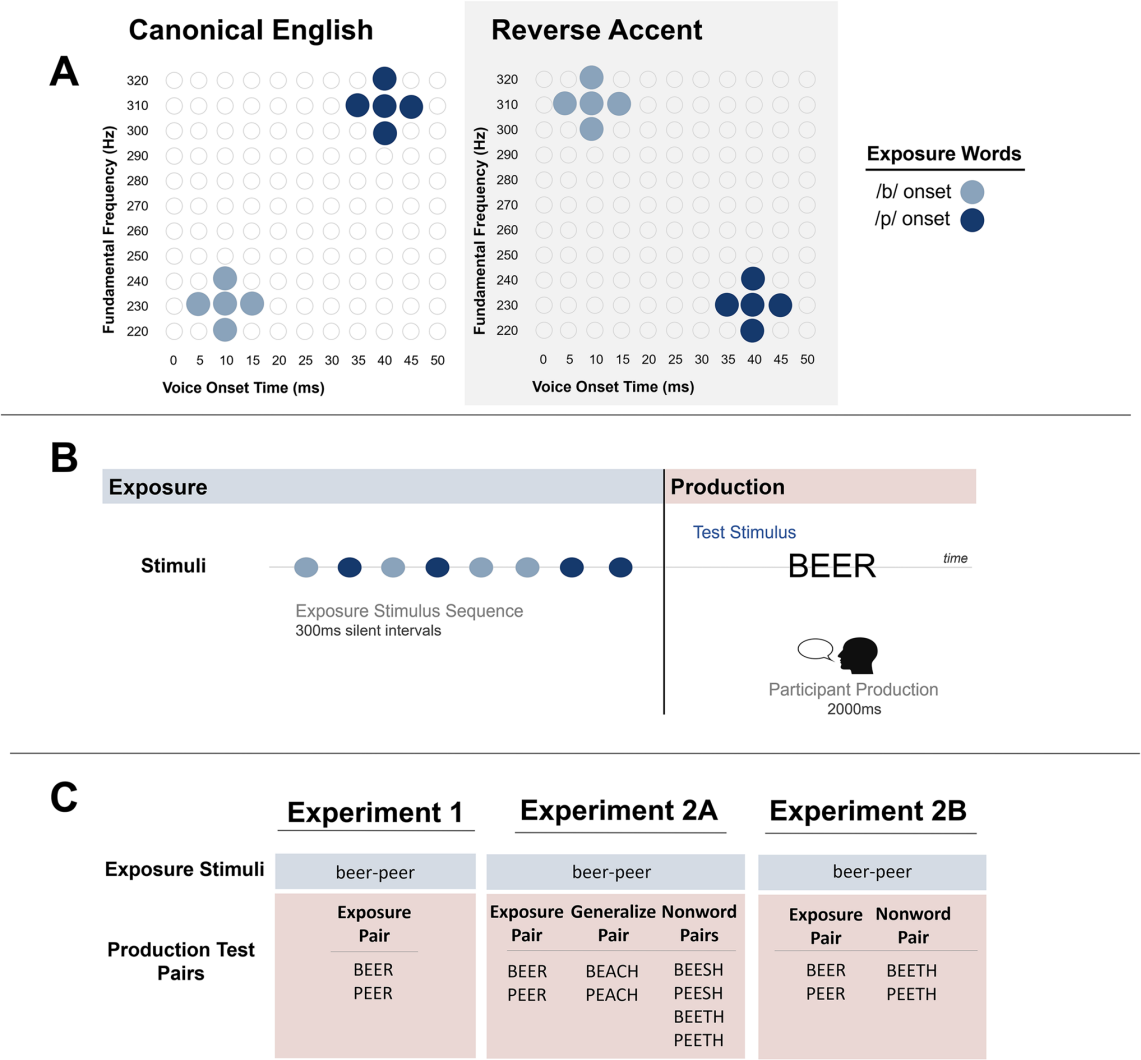
Outline of Experimental Design. **A. **Distribution of stimuli. Exposure stimuli are distributed across an acoustic space of voice onset time (VOT) and fundamental frequency (F0). Conditions are defined by a subsampling of this space that matches American English speech regularities (Canonical) or for which the VOT x F0 correlation is Reversed to create a subtle accent. **B. **Trial procedure. Participants heard eight words randomly sampled from the condition’s stimulus distribution, with equal sampling from short- and long-VOT regions, and then read aloud a printed word **C.** Stimulus pairs in Experiments 1, 2A and 2B. In all experiments, participants heard beer-peer in exposure. Production Test Pairs included BEER-PEER, the lexical-generalization pair BEACH-PEACH, and the nonword generalization pairs BEESH-PEESH and BEETH-PEETH

Materials consisted of auditory stimuli for the exposure phase and written words for the test phase. Exposure stimuli were audio recordings of the words *beer* and *peer* adopted from Murphy et al. (2025b). The specific tokens for *beer* and *peer* had a similar duration (400 ms) and F0 contour*.* Fundamental frequency (F0) at the onset of voicing was manipulated along with VOT to create a two-dimensional F0 × VOT acoustic space. F0 onset frequency was manipulated in 10-Hz steps in a 220- to 320-Hz range, and VOT was manipulated in 5-ms steps in a 5- to 40-ms range. Auditory stimuli were sampled from this two-dimension acoustic space to create two statistical regularity conditions: Canonical and Reverse. Figure 1A displays the distribution of stimuli in the two conditions. In the Canonical condition, which simulates the American English F0 × VOT relationship, /b/ was represented with a low F0 range of 220–240 Hz and a short VOT range of 5–15 ms and /p/ was represented with a high F0 range of 300–320 Hz and a long VOT range of 35–45 ms. In the Reverse condition, which simulated an accent, this mapping was flipped: stimuli with short VOTs were placed in the high F0 range (heard as *beer*), and stimuli with long VOTs (heard as *peer*) were placed in the low F0 range. We sampled multiple points in the F0 × VOT space allotted for each condition, allowing us to simulate variability in speech input while preserving the overall F0 × VOT correlation. Test stimuli were words BEER and PEER printed in uppercase black Calibri font (78 pt) on a white background.

#### Procedure

Online participants were recruited via Prolific and directed to the experiment hosted on Gorilla (www.gorilla.sc; Anwyl-Irvine et al., 2020). Participants completed consent and demographics forms before performing a headphone check (Milne et al., 2021) and a microphone check. Those who did not pass the headphone check after two attempts were rejected from the experiment. Next, participants read instructions and watched a demo video of a sample trial with the orthographic test syllable BEEV, which was not presented in the experiment.

Each trial consisted of an auditory exposure phase and a word reading test phase. Figure 1B depicts the trial structure. In the auditory exposure phase, participants heard eight auditory stimuli randomized in order across trials, with four having a short VOT and four having a long VOT heard by most listeners as *beer* and *peer,* respectively. Immediately after, in the test phase, one target word appeared on-screen and the participant read it aloud within a 2,000-ms deadline before moving on to the next trial. Participants’ recordings were saved as.weba files onto Gorilla’s servers. The experiment consisted of 64 trials, divided into four equal blocks of 16 trials. Blocks alternated between Canonical and Reverse conditions, such that Blocks 1 and 3 used the Canonical stimuli, and Blocks 2 and 4 used the Reverse stimuli. Following Murphy et al. (2025a, 2025b), we chose to lead with a Canonical block to allow us to introduce the accent in the Reverse block as a shift from English expectations. Orthographic test stimuli were identical across blocks. Every eight trials, there was a 15-s break. Two lists were created with randomized order of trials. Each participant was randomly assigned to one of the two lists. The experiment took approximately 15 min to complete.

#### Analysis

Acoustic speech analysis was conducted using a custom pipeline described in Murphy et al. (2024). Trials with an incorrect or unclear response, disfluencies, murmuring, or too much background noise were excluded from further analysis, as they do not provide reliable F0 measures. Acoustic speech analysis was conducted using a custom pipeline described in Murphy et al. (2024), using Praat (version 6.4.23; Boersma & Weenink, 2024). First, “To TextGrid (silences)...” identified and isolated word productions in the 2.5-s audio recordings. Next, “To Pitch (ac)” measured the F0 frequency of the first 40 ms of voicing, where F0 differences between onset obstruent consonants are typically most pronounced (Hanson, 2009; Hombert et al., 1979; Lea, 1973; Xu & Xu, 2021). F0 measurements were made in 10-ms intervals, resulting in five samples per recording, which were averaged to generate one measurement per trial. Measurements that were ±3 standard deviations from the participant’s average F0 across all productions were excluded from analysis. F0 was then normalized on a by-individual basis to mitigate the effects of between-subject variation in F0, such as sex (Titze, 1989). Thus, a *z* score of 0 represents the mean F0 for a participant across all their productions, and positive and negative *z* scores indicate higher or lower F0 relative to their mean, in standard deviation units. These *z* scores were the dependent measure of our analyses.

In general, we used generalized multi-level models with mixed effects whenever applicable. The random effect structure in Experiment 1 would have contained a random intercept of subjects, as well as random slopes for fixed effects over subjects. However, the model’s estimation of random slopes was close to zero. Dropping random slopes would make the model similar to analysis of variance (ANOVA). Therefore, for this experiment, we used a 2 × 2 ANOVA with Condition (Canonical vs. Reverse) and First Letter (B vs. P) as fixed factors. ANOVA was performed with the *afex* package (Version 1.4.1; Singmann et al., 2024) in R (Version 4.5.0; R Core Team, 2025). The data and results for all experiments are available on the Open Science Framework (OSF).

### Results

**Fig. 2:**
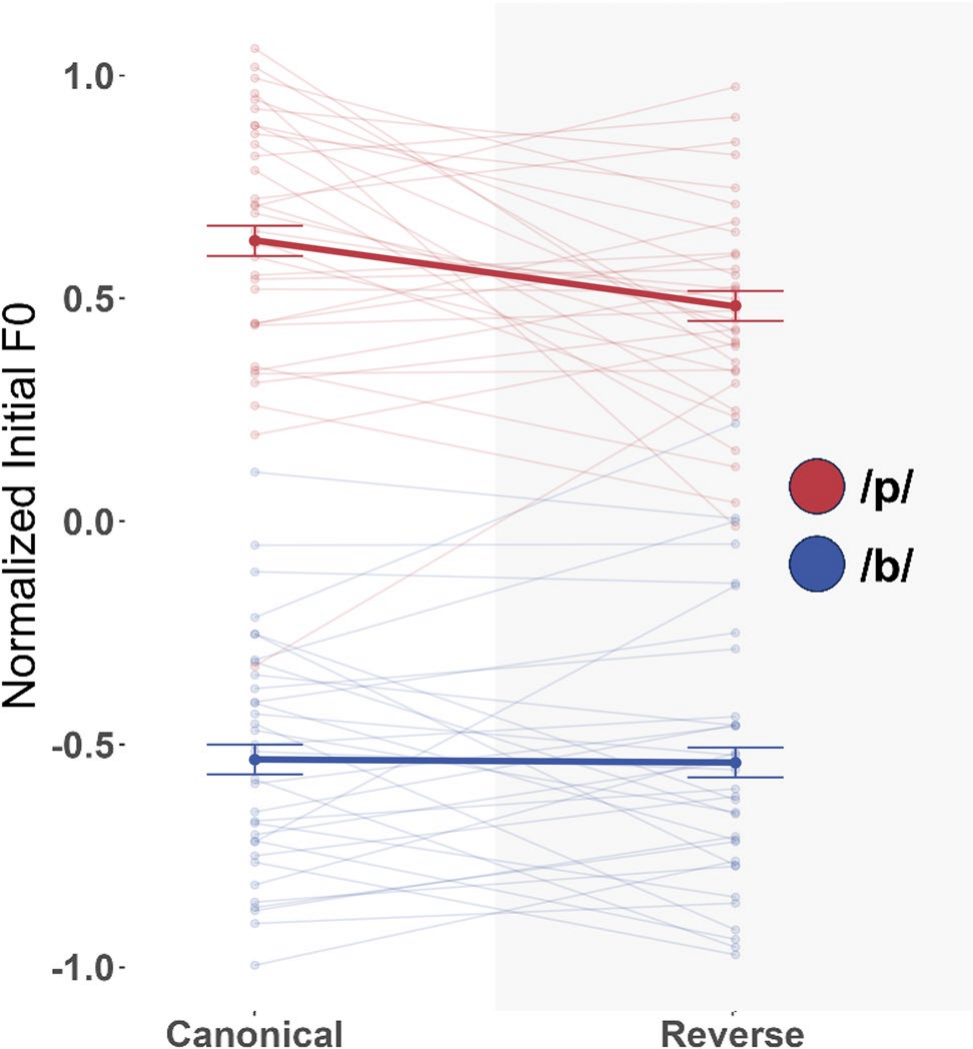
Experiment 1 results. Shows z score normalized initial F0 for reading-elicited production of beer (blue) and peer (red) across both conditions. The thick line indicates the sample mean; transparent lines show individual subjects’ means. Error bars indicate ±1 standard error of the mean across subjects

In total, 4.88% of responses were excluded, with similar exclusion rates across the Canonical and Reverse condition. Figure 2 displays the results (see Table A1 in the Online Supplementary Material for descriptive statistics) and Table 1 presents the results of ANOVA.

**Table 1:** The 2 × 2 ANOVA of Experiment 1

Measure	*MSE*	*F(1, 32)*	Gη^2^	*p*
Condition	0.07	2.88	.020	.099
First Letter	0.20	192.92	.796	<.001
Condition × First Letter	0.02	8.63	.016	.006

As expected, F0 was significantly higher in PEER than BEER, *F*(1, 32) = 192.92, *p* <.001. There was also a marginal main effect of Condition, *F*(1, 32) = 2.88, *p* =.099. Importantly for our purpose, there was a significant interaction between First Letter and Condition, *F*(1, 32) = 8.63, *p* =.006.

### Discussion

Experiment 1 demonstrated that the statistics of the incoming auditory signal affect the production of written forms, without an auditory probe. Experiment 2 A tested the generalization of this effect to lexical and nonlexical pairs.

## Experiment 2A

### Methods

#### Participants

A power analysis (PANGEA; Westfall, 2015) showed that a sample size of 39 was required to detect a three-way Condition × First Letter × Pair interaction with an effect size of 0.2 as the smallest effect size of interest, with a power of 0.85 and alpha of 0.05. Given the number of participants who had to be excluded due to background noise in Experiment 1, we recruited a larger sample of 65 participants for Experiment 2A. Exclusion criteria and compensation were similar to Experiment 1. After exclusions, data from 50 participants (*M*_Age_ = 26.87, *SD* = 6.14 years; *N*_Female_ = 28) remained.

#### Stimuli

The Experiment 1 acoustic speech stimuli were used for the exposure phase. Test stimuli comprised four pairs. BEER-PEER was a lexical pair identical to the exposure stimuli (Word-Exposure), used to replicate the results of Experiment 1. BEACH-PEACH was a lexical pair different from the exposure stimuli, suitable for assessing generalization to other words that share a common onset with the exposure pair (Word-Generalization). BEESH-PEESH and BEETH-PEETH were nonlexical pairs, and thus obviously different from the exposure stimuli, suitable for assessing generalization to nonwords that share a common onset with the exposure pair (Nonword-Generalization). Two nonlexical pairs were used to balance the frequency of words and nonwords in the experiment, because different ratios of words and nonwords can change how participants read words (e.g., Hartsuiker et al., 2005). As such, each test pair consisted of 25% of the total trials. All words appeared on-screen in uppercase 78-pt black Calibri font on a white background.

#### Procedure

The design was similar to that of Experiment 1. Experiment 2A consisted of 256 trials, with two blocks divided equally into 128 trials. There was a 15-s break every eight trials, with a longer 60-s break every 32 trials. Block 1 was Canonical, and Block 2 was Reverse. Within each block, each of the eight possible test words appeared 16 times. Three orders were created with a pseudorandomized trial order, with the following constraints: (1) words in the same test pair could not appear in subsequent trials, (2) no more than three words in a row could start with the same first letter, and (3) the lists were evenly divided into four quarters and each test word appeared a total of eight times in each quarter. Participants were randomly assigned to one of the three orders. Participants completed the same trial procedure as before. The experiment took approximately 50 min to complete.

#### Analysis

We used the same F0 extraction and normalization procedure as in Experiment 1. Data were analyzed using linear mixed-effect models implemented in the *lme4* package with the lmer function (Bates et al., 2015) in R (Version 4.5.0; R Core Team, 2025). The dependent variable was normalized F0. Fixed effects included Condition (Canonical vs. Reverse) and First Letter (B vs. P). To measure generalization, we included two additional factors: Pair (BEACH-PEACH vs. BEER-PEER) to evaluate transfer to novel lexical items, and Lexicality (Word vs. Nonword) to test generalization from words to nonwords. All fixed effects were sum-coded (−1, 1). *P* values were calculated using Satterthwaite approximations via the *lmerTest* package (Version 3.1.3; Kuznetsova et al., 2017). We used the largest random effect structure that could be tolerated by each model.

Three sets of analyses were conducted on the data. Set 1 was a general analysis to test for the downweighting of F0 across all four pairs. The model had normalized initial Production F0 as its dependent variable. The fixed-effect structure included Condition (Canonical vs. Reverse) and First Letter (B vs. P), as well as the two-way interaction between them. The random-effect structure included the random intercept of subjects, as well as the random slopes of Condition and First Letter over subjects.

Set 2 examined lexical generalization. Correspondingly, it used the subset of data with lexical pairs (BEER-PEER and BEACH-PEACH). The model had normalized initial Production F0 as its dependent variable. The fixed-effect structure included Condition (Canonical vs. Reverse), First Letter (B vs. P), Pair (BEER-PEER vs. BEACH-PEACH), as well as all the two- and three-way interactions between them. The random-effect structure included the random intercept of subjects, as well as the random slopes of Condition, First Letter, and Pair over subjects. We followed up on this analysis by reporting the results of two post hoc tests conducted individually on BEER-PEER and BEACH-PEACH subsets to investigate F0 downweighting in each pair individually.

Finally, Set 3 examined generalization to nonlexical items. Given the results of Set 2, which found very similar patterns between BEER-PEER and BEACH-PEACH, the final set included all four pairs in order to have the most balanced analysis. This model had normalized initial production F0 as its dependent variable. The fixed-effect structure included Condition (Canonical vs. Reverse), First Letter (B vs. P), Lexicality (Word vs. Nonword), as well as the two- and three-way interactions between them. The random-effect structure included the random intercept of subjects, as well as the random slopes of Condition, First Letter, and Lexicality over subjects. We followed this analysis with a post hoc test on BEESH-PEESH and BEETH-PEETH combined to investigate downweighting in nonlexical pairs.

### Results

**Fig. 3:**
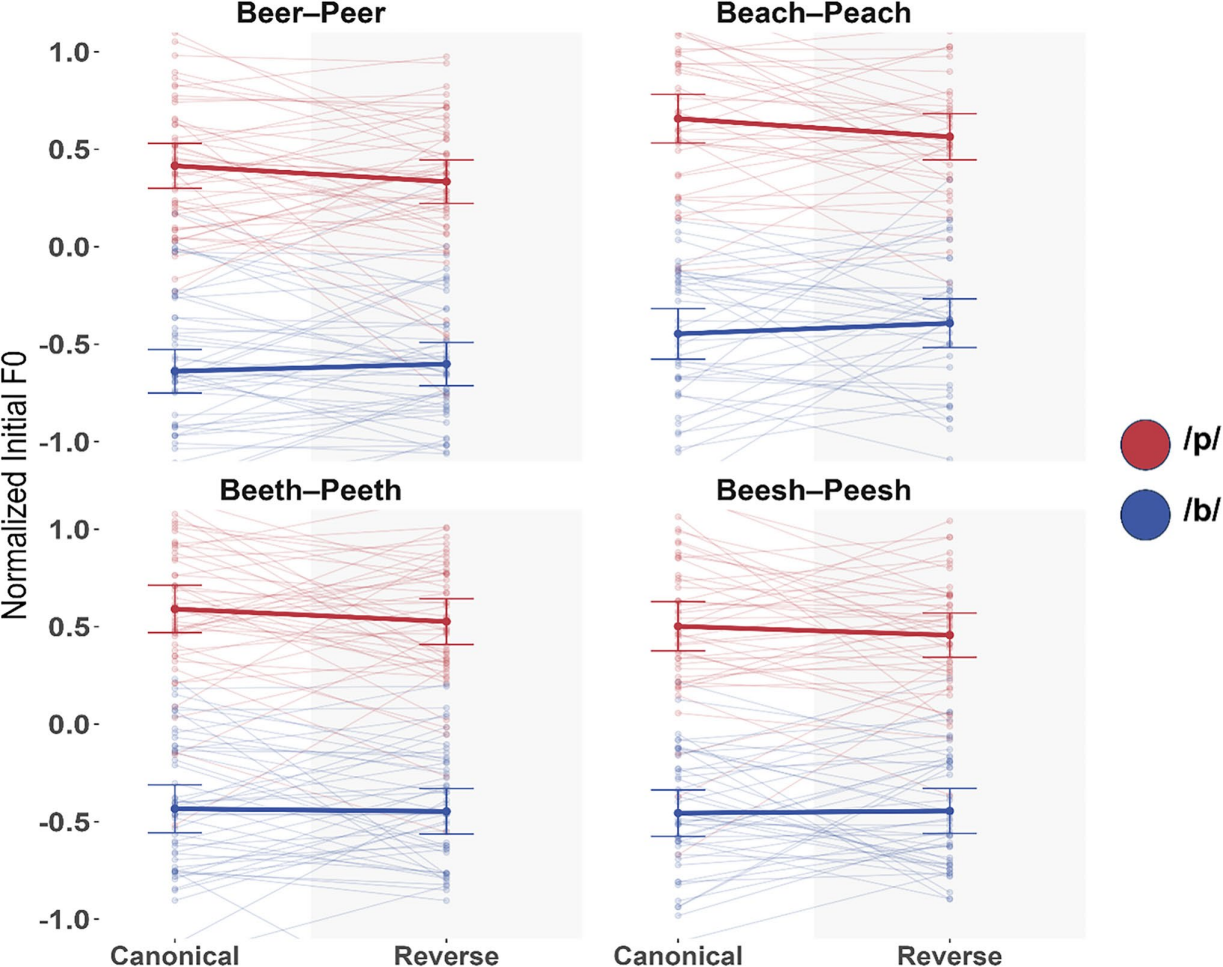
Experiment 2 A results. Shows z score normalized initial F0 for participant production of /b/ (blue) and /p/ (red) across both conditions in all four test pairs. The thick line indicates the sample mean; transparent lines show individual subjects’ means. Error bars indicate ±1 standard er*ror of the mean across subjects*

In total, 13.63% of trials were excluded due to erroneous, unclear, or noisy productions, with similar exclusion rates across the Canonical and Reverse conditions. Figure 3 shows the results of Experiment 2 A (see Table A1 in the Online Supplementary Material for descriptive statistics).

*Set 1*. Table 2 shows the results of the general analysis. Across all trials, F0 was significantly higher in /p/ utterances than /b/ utterances (β = −0.4864, *t* = −15.067, *p* <.001). The main effect of Condition was not significant (β = 0.0136, *t* = 0.752, *p* =.455). Importantly for our purpose, there was a significant interaction between First Letter and Condition across all trials (β = -.02449, *t* = −3.542, *p* <.001).

**Table 2:** Results of the Set 1 analysis for Experiment 2A

Predictor	β	*SE*	*t*	*p*
Intercept	0.003	0.010	0.296	.768
Condition	0.014	0.018	0.752	.455
First Letter	−0.486	0.032	−15.067	<.001
Condition × First Letter	−0.025	0.007	−3.542	<.001

*Set 2.* Table 3 shows the results of the lexical-generalization analysis. As expected, F0 was significantly higher for /p/ than for /b/ (β = −0.499, *t* = −15.837, *p* <.001). There was also a main effect of Pair, with F0 overall higher in BEACH-PEACH than in BEER-PEER (β = 0.109, *t* = 5.163, *p* <.001). Importantly for our purpose, there was a significant interaction between Condition and First Letter, marking F0 downweighting (β = −0.034, *t* = −3.472, *p* <.001). However, downweighting did not interact with Pair (β = −0.004, *t* = −0.444, *p* =.657). Follow-up tests showed a significant main effect of First Letter for both BEER-PEER and BEACH-PEACH pairs (BEER-PEER: (β = −0.494, *t* = −14.802, *p* <.001); BEACH-PEACH: (β = −0.519, *t* = −13.945, *p* <.001). Importantly, they showed a significant Condition × First letter interaction in BEER-PEER (β = –0.030, *t* = –2.430, *p* =.015), replicating the results of Experiment 1, and a similar interaction in BEACH-PEACH (β = –0.037, *t* = –2.380, *p* =.017), showing generalization to a new lexical pair.

**Table 3:** Results of the Set 2 (lexical generalization) analysis for Experiment 2A

Predictor	β	*SE*	*t*	*p*
Intercept	−0.014	0.015	−0.956	.344
Condition	0.013	0.018	0.726	.471
First Letter	−0.499	0.031	−15.837	<.001
Pair	0.109	0.021	5.163	<.001
Condition × First Letter	−0.034	0.010	−3.472	<.001
Condition × Pair	0.001	0.010	0.048	.961
First Letter × Pair	−0.005	0.010	−0.527	.598
Condition × First Letter × Pair	−0.004	0.010	−0.444	.657

*Set 3.* Table 4 shows the results of the nonlexical-generalization analysis. Because of similar effect sizes demonstrated by the post hoc analyses in Set 2, we chose to include BEACH-PEACH trials in this model to maintain symmetry between factors. As expected, F0 was significantly higher for /p/ than /b/ (β = −0.487, *t* = −15.062, *p* <.001). There was also a main effect of Lexicality, meaning that F0 was overall higher in BEESH-PEESH and BEETH-PEETH than in BEER-PEER and BEACH-PEACH (β = 0.038, *t* = 3.117, *p* =.003). There was a significant interaction between Condition and First Letter (β = −0.025, *t* = −3.610, *p* <.001), replicating the transfer effect across the full dataset. Overall downweighting did not interact with Lexicality (β = 0.009, *t* = 1.275, *p* =.202), suggesting that the change in participant F0 from Canonical to Reverse conditions was similar for both Words and Nonwords. A follow-up test on the subset of all nonlexical trials showed a significant main effect of First Letter (β** =** −0.470, *t* = −13.12, *p* <.001). However, there was a marginal Condition × First Letter interaction (β = −0.016, *t* = −1.68, *p* = 0.093). While the overall model suggests that transfer may extend to Nonwords, the marginal subset result leaves this conclusion uncertain.

**Table 4:** Results of the Set 3 (nonlexical generalization) analysis for Experiment 2A

Predictor	β	*SE*	*t*	*p*
Intercept	−0.000	0.011	−0.031	.975
Condition	0.014	0.018	0.759	.451
First Letter	−0.487	0.032	−15.062	<.001
Lexicality	0.038	0.012	3.117	.003
Condition × First Letter	−0.025	0.007	−3.610	<.001
Condition × Lexicality	0.001	0.007	−0.083	.933
First Letter × Lexicality	0.012	0.007	1.675	.094
Condition × First Letter × Lexicality	0.009	0.007	1.275	.202

### Discussion

In summary, Experiment 2A replicated the results of Experiment 1, and showed clear generalization to a new lexical pair with a magnitude comparable to that of the original pair. However, while there was no significant interaction between downweighting and lexicality, downweighting in the combined set of nonlexical pairs was only marginal, preventing us from drawing clear conclusions about the scope of generalization. There are two possibilities for the marginal effect of nonlexical generalization. First, generalization may be weak or unstable for nonlexical pairs. Second, generalization may be solid for such pairs, but the data may have been too noisy to detect it. The latter is a distinct possibility, as the inclusion of 16 syllables in a much longer experiment led to many more exclusions due to errors and unclear productions, compared to Experiment 1. One pair, BEESH-PEESH, was specifically error-prone, generating 39% of errors made across all four pairs. We thus designed Experiment 2B as a shorter version of Experiment 2A, focusing on contrasting BEER-PEER with the cleaner nonword pair BEETH-PEETH. If there is generalization to nonlexical pairs, we expect Experiment 2B to show such generalization clearly.

## Experiment 2B

### Methods

#### Participants

Given the similarity in the goals of Experiments 2A and 2B, the same sample size calculation was employed, requiring 39 subjects. We recruited 53 subjects, and used 43 after excluding those who did not follow task instructions or had noisy backgrounds (*M*_Age_ = 30.11, *SD* = 4.18 years; *N*_Female_ = 22).

#### Stimuli

The materials were the same as in Experiment 2A but only included BEER-PEER and the nonword BEETH-PEETH.

#### Procedure

The procedure matched Experiments 1 and 2A. Experiment 2B consisted of 128 trials, with two equal blocks of 64 trials each. Block 1 was the Canonical condition, and Block 2 was the Reverse condition. In each block, each of the four possible test words appeared 16 times. For the experiment, two test orders were created. Each order was split into equal quarters, and each test word was used an equal number of times per quarter. Participants were randomly assigned to an order. The experiment took approximately 25 min to complete.

#### Analysis

The same analysis as Set 3 of Experiment 2A was applied here. The model had normalized F0 as its dependent variable. The fixed-effect structure included Condition (Canonical vs. Reverse), First Letter (B vs. P), and Lexicality (Word vs. Nonword) as well as the two- and three-way interactions between them. The random-effect structure included the random intercept of subjects, as well as the random slopes of Condition and First Letter over subjects. We followed up on this analysis by conducting a post hoc test on both BEER-PEER and BEETH-PEETH.

### Results

**Fig. 4:**
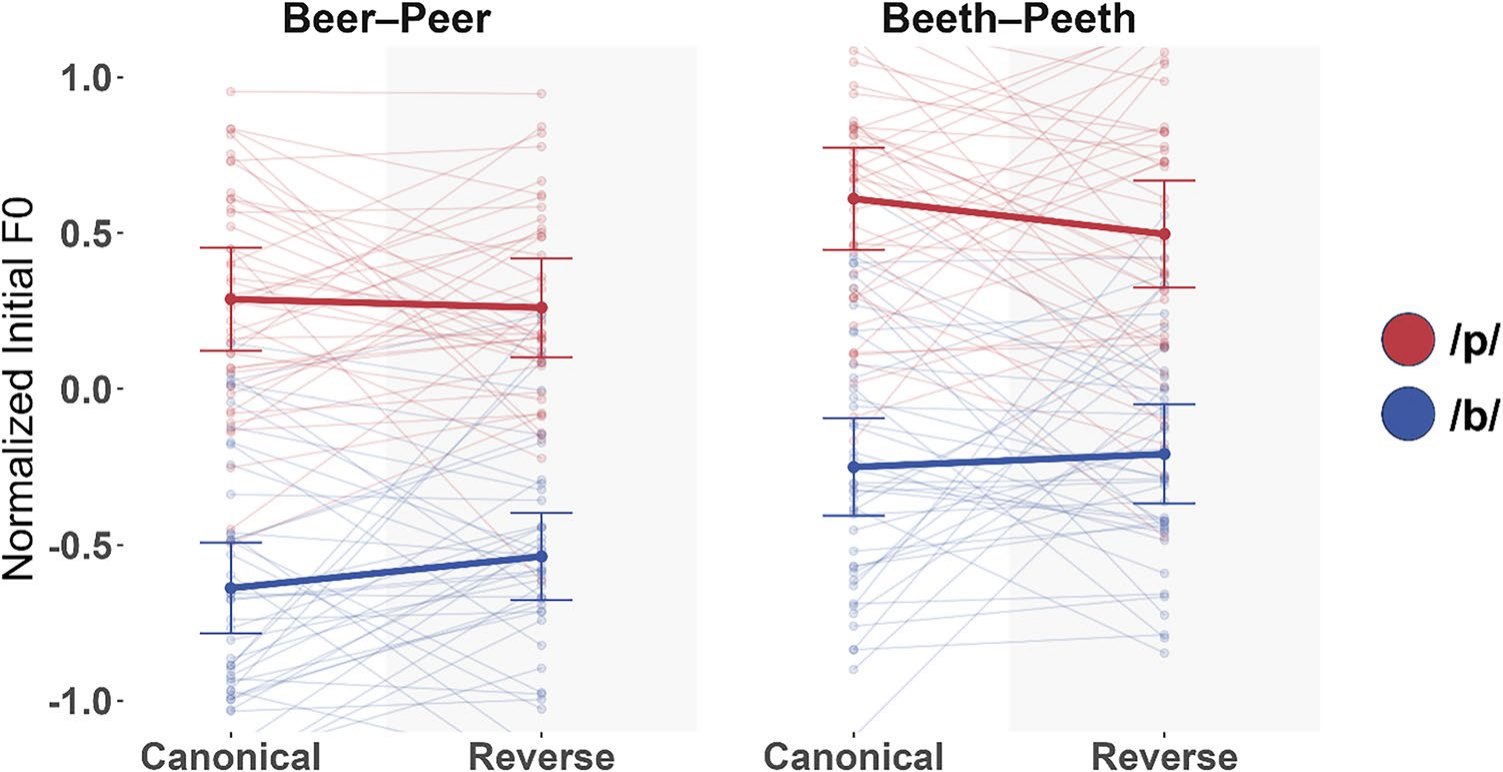
Experiment 2B results. score normalized initial F0 for participant production of /b/ (blue) and /p/ (red) across both conditions. The thick line indicates the sample mean; transparent lines show individual subjects’ means. Error bars indicate ±1 standard error of the mean across subjects.

In total, 4.09% of trials were excluded. In the Canonical condition, 3.99% of trials were excluded. In the Reverse condition, 4.17% of trials were excluded. Figure 4 shows the pattern of results in Experiment 2B and Table 5 summarizes the results of the analysis. As before, we found significantly higher F0s for /p/ versus /b/ (β = −0.412, *t* = −11.548, *p <*.001). F0 was also significantly higher for BEETH-PEETH than BEER-PEER (β = 0.159, *t* = 14.569, *p* <.001). Importantly, we saw a significant interaction between Condition and First Letter, denoting downweighting (β = −0.035,* t* = −3.224, p =.0012), which did not interact with Lexicality (β = −0.003, *t* = −0.267, *p* =.789).

**Table 5:** Lexicality model results for Experiment 2B

**Predictor**	**β**	***SE***	***t***	***p***
Intercept	0.002	0.011	0.181	.856
Condition	−0.001	0.031	−0.046	.963
First Letter	−0.412	0.036	−11.548	<.001
Lexicality	0.159	0.011	14.569	<.001
Condition × First Letter	−0.035	0.011	−3.224	.001
Condition × Lexicality	0.016	0.011	1.485	.138
First Letter × Lexicality	0.020	0.011	1.855	.064
Condition × First Letter × Lexicality	−0.003	0.011	−0.267	.789

Post hoc tests showed higher F0 for /p/ versus /b/ in both BEER-PEER and BEETH-PEETH (BEER-PEER: β = −0.432, *t* = −12.084, *p* <.001; BEETH-PEETH: β = −0.394, *t* = −9.716*, p* <.001). Critically, they also showed a significant interaction between Condition and First Letter for BEER-PEER (β = −0.031, *t* = −2.093, *p* =.0365), providing a third replication of F0 downweighting in reading, as well as BEETH-PEETH (β = −0.038, *t* = −2.447, *p* =.0145), showing clear generalization to a nonlexical pair.^1^

## General discussion

The results of Experiment 1 show that the perception-production transfer previously reported in auditory repetition tasks (Murphy et al., 2024, 2025a, 2025b) extends to reading. We replicated this effect twice more in Experiments 2A and 2B, leaving no room for doubt that an auditory prompt was not necessary for changes to the production system after exposure to new statistics in the perception system. We further showed generalization to new pairs that had never been auditorily modeled in the study. Based on past studies that had limited generalization in perception to shared phonemes (Idemaru & Holt, 2014, 2020; Murphy et al., 2025b), we calibrated our generalization experiments to this level and examined generalization to both words and nonwords.

Experiment 2 A found generalization to a new word pair. Moreover, the magnitude of the transfer effect in the new pair was comparable to that of the exposure pair, suggesting that a shared CV, rather than the full syllable (CVC), is sufficient to produce generalization. In contrast, only a marginal generalization effect was obtained for the nonlexical pair in this experiment. As this result did not match either of the two theoretical possibilities discussed in the Introduction, we surmised that the marginal effect may have stemmed from a low signal-to-noise ratio. Indeed, one of the two nonlexical pairs (BEESH-PEESH) was often read with errors and disfluencies, leading to the exclusion of a large number of trials. Given that the initial F0 is already quite variable in production (Murphy et al., 2024), exclusion of too many trials can introduce substantial noise into the analyses. Experiment 2B examined this possibility by focusing on the cleaner nonlexical pair (BEETH-PEETH). This experiment replicated the generalization observed in Experiment 2A, but this time with the nonlexical pair. Also similar to Experiment 2A, the size of the transfer effect was comparable between the exposure and the nonword pair, identifying the phoneme (and not the full CVC syllable) as the critical representation involved in transfer.

To summarize, we found perception-production transfer in reading that generalized readily to both new lexical and nonlexical stimuli that shared the critical phoneme with the exposure pair. Contrary to the prediction of a GPC-driven process, generalization was not weaker for words than nonwords. This finding implies that the change to the production system affects post-lexical (most likely articulatory-phonetic) representations, regardless of how they are accessed (lexically or sublexically). This is important for extending the scope of transfer from laboratory studies to everyday conversations. If transfer were limited to the sublexical route, it would significantly limit the possibility of observing transfer in more naturalistic contexts, where articulation is driven primarily by lexical access.

The uncovering and replication of generalization in the current study is different from the null generalization effect in production reported in Murphy et al. (2025b). The most likely reason for this difference is that the unit of generalization in production is the CV (current study) rather than the phoneme (Murphy et al., 2025b). This interpretation is supported by studies emphasizing the unique role of syllables in production during phonetic encoding (e.g., Cholin et al., 2006; Laganaro & Alario, 2006). Although we cannot, based on current results, rule out a role for task in explaining the differences in generalization between the current study and Murphy et al. (2025b), there is no clear theoretical reason why task should affect generalization, especially when the basic effect was robustly replicated across tasks. Nevertheless, we propose that future studies compare generalization at similar levels across tasks within the same individuals to establish the robustness and scope of generalization.

## Supplementary Information

Below is the link to the electronic supplementary material.

Supplementary file1 (DOCX 20 KB)

## Data Availability

The data and tables used in the study are available via the Open Science Framework at: https://osf.io/kusr4/?view_only=47488caa1de14739a87afec68a380d5b

## Footnotes

1 See Appendix B in the Online Supplementary Material for a set of cross-experiment analyses testing the influence of pure lexical versus mixed lexical-nonlexical contexts on word reading.

## Code availability

The scripts used for statistical analyses in the study are available via the Open Science Framework at: https://osf.io/kusr4/?view_only=47488caa1de14739a87afec68a380d5b
