# Supplementary material for: Transfer of statistical learning from speech perception to production generalizes to reading
Source: Psychon Bull Rev. 2026 Mar 4;33(3):104. doi: 10.3758/s13423-026-02874-y (PMC12960428; doi:10.3758/s13423-026-02874-y)
Supplement: Supplementary file 1 — Supplementary file1 (DOCX 20 KB) [file 13423_2026_2874_MOESM1_ESM.docx]

**Appendix A: Descriptive statistics across experiments**

Table A1. Mean normalized F0 (in *z* scores) for Canonical and Reverse conditions in each experiment, along with the within-subject shift in F0, computed as the difference between Canonical and Reverse conditions. Standard errors (in parentheses) are computed over subject-level means.

| *Experiment* | *Participants* | *Canonical (SE)* | *Reverse (SE)* | *Shift (SE)* |
| --- | --- | --- | --- | --- |
| Exp 1 | 33 | 1.155 (0.083) | 1.018 (0.079) | 0.137 (0.046) |
| Exp 2A | 50 | 1.021 (0.071) | 0.915 (0.063) | 0.106 (0.036) |
| Exp 2B | 43 | 0.895 (0.080) | 0.756 (0.071) | 0.139 (0.051) |

**Appendix B: Cross-experiment analyses**

As alluded to earlier, different ratios of words and nonwords could change how participants read words (e.g., Hartsuiker et al., 2005). In Experiment 1, participants were only exposed to words, whereas in Experiments 2A and 2B, they were exposed to a balanced mixture of words and nonwords. It is thus possible that participants have treated the word pairs as nonwords in the latter two experiments. This, in turn, could change the size of transfer for word pairs. To investigate this issue, we have selected the common subset of all three experiments, i.e., “BEER-PEER” pairs, and statistically compared the size of transfer for this pair in Experiment 1 vs. Experiment 2A (Table B1) and Experiment 2B (Table B2). Models were similar in structure to those described in detail in the main text, with the exception of the added variable of “Context” (Pure lexical vs. Mixed lexical-nonlexical) to the fixed structure and its interactions with the other two fixed effects, Condition and First Letter. The models’ random effect structure included the random intercept of subjects, as well as the random slopes of Condition and First Letter.

Table B1. BEER-PEER downweighting compared between Experiment 1 and Experiment 2A.

| *Predictor* | β | *SE* | *t* | *p* |
| --- | --- | --- | --- | --- |
| Intercept | -0.055 | 0.017 | -3.267 | .002 |
| Condition | 0.027 | 0.015 | 1.755 | .083 |
| First Letter | -0.519 | 0.026 | -20.049 | < .001 |
| Context | 0.068 | 0.017 | 4.045 | < .001 |
| Condition × First Letter | -0.032 | 0.010 | -3.219 | .001 |
| Condition × Context | 0.015 | 0.015 | 0.971 | .334 |
| First Letter × Context | -0.025 | 0.026 | -0.968 | .336 |
| Condition × First Letter × Context | -0.002 | 0.010 | -0.201 | .840 |

Table B2. BEER-PEER downweighting compared between Experiment 1 and Experiment 2B.

| *Predictor* | β | *SE* | *t* | *p* |
| --- | --- | --- | --- | --- |
| Intercept | -0.072 | 0.015 | -4.961 | < .001 |
| Condition | 0.012 | 0.020 | 0.613 | .542 |
| First Letter | -0.488 | 0.027 | -18.346 | < .001 |
| Context | 0.085 | 0.015 | 5.844 | < .001 |
| Condition × First Letter | -0.033 | 0.011 | -2.963 | .003 |
| Condition × Context | 0.029 | 0.020 | 1.482 | .142 |
| First Letter × Context | -0.056 | 0.027 | -2.111 | .038 |
| Condition × First Letter × Context | -0.001 | 0.011 | -0.124 | .902 |

In both analyses, there was a main effect of Context, with average F0 higher in Experiment 1 compared to both Experiments 2A and 2B, most likely reflecting the differences in the samples of these experiments. However, the effect of interest to us is the three-way interaction between Condition x First Letter and Context. This interaction was nowhere near significant in either analysis. To make sure that this null statistical result was not due to low statistical power, we conducted a third analysis, aggregating the data from Experiments 2A and 2B (this aggregation was sanctioned by the similar pattern observed in the two analyses reported above). The results are reported in Table B3.

Table B3. BEER-PEER downweighting compared between Experiment 1 and the aggregate BEER-PEER responses across Experiments 2A and 2B.

| *Predictor* | β | *SE* | *t* | *p* |
| --- | --- | --- | --- | --- |
| Intercept | -0.063 | 0.015 | -4.098 | < .001 |
| Condition | 0.020 | 0.016 | 1.249 | .214 |
| First Letter | -0.505 | 0.024 | -21.404 | < .001 |
| Context | 0.076 | 0.015 | 4.944 | < .001 |
| Condition × First Letter | -0.033 | 0.009 | -3.472 | < .001 |
| Condition × Context | 0.021 | 0.016 | 1.335 | .184 |
| First Letter × Context | -0.039 | 0.023 | -1.671 | .097 |
| Condition × First Letter × Context | -0.002 | 0.009 | -0.185 | .853 |

As can be seen in the table, the three-way interaction remained non-significant. We can, thus, conclude that the lexicality of context did not significantly modulate the size of the transfer effect for lexical items.
